# A pilot oral history of plant synthetic biology

**DOI:** 10.1093/plphys/kiad585

**Published:** 2024-01-02

**Authors:** Jaya Joshi, Andrew D Hanson

**Affiliations:** Department of Wood Science, University of British Columbia, 2424 Main Mall, Vancouver, BC V6T 1Z4, Canada; Horticultural Sciences Department, University of Florida, 2550 Hull Road, Gainesville, FL 32611, USA

## Abstract

The whole field of synthetic biology (SynBio) is only about 20 years old, and plant SynBio is younger still. Nevertheless, within that short time, SynBio in general has drawn more scientific, philosophical, government, and private-sector interest than anything in biology since the recombinant DNA revolution. Plant SynBio, in particular, is now drawing more and more interest in relation to plants’ potential to help solve planetary problems such as carbon capture and storage and replacing fossil fuels and feedstocks. As plant SynBio is so young and so fast-developing, we felt it was too soon to try to analyze its history. Instead, we set out to capture the essence of plant SynBio's origins and early development through interviews with 8 of the field's founders, representing 5 countries and 3 continents. We then distilled these founders’ personal recollections and reflections into this review, centering the narrative on timelines for pivotal events, articles, funding programs, and quoting from interviews. We have archived the interview recordings and documented timeline entries. This work provides a resource for future historical scholarship.

## Teaser

Plant synthetic biology is a rising field that can help agriculture, industry, and the environment.

## Introduction

### What synthetic biology is and does

Synthetic biology (SynBio) has been defined in various ways, of which these 2 are typical: “[SynBio] aims to build new properties into living systems so that they do or make something useful” (Davies 2019); and “SynBio is a transformative combination of DNA technology, engineering principles, and computational tools that makes it possible to design new life processes and to repurpose existing natural ones for useful purposes” (Hanson et al. 2019). These and most other definitions stress that SynBio designs, builds, and uses biological components (“parts”) that do not exist in nature, and that the end goal is utility, i.e. that at its core, SynBio is primarily an engineering endeavor that aims to solve problems, not a scientific one that seeks to answer questions and to uncover information. To understand SynBio it is therefore essential to grasp the differences between engineering and science. The following pithy quotes—and the articles they come from—sum up key characteristics of engineering:

Scientists study the world as it is, engineers create the world that never has been (Hammack and Anderson 2022);

The engineering method is the use of heuristics to cause the best change in a poorly understood situation within the available resources (Hammack and Anderson 2022);

Critically different from colleagues in the sciences, the chemical engineer must be willing to attempt a solution in situations in which many important details of the system are undefined or uncertain (Bailey 1995);

In order to achieve reliable [engineering] outcomes it is necessary to avoid jumping to conclusions, to avoid making uninformed guesses where information can be made available. (MacLeod 2012)

The last quote refers particularly to the value of numerate thinking, which emerged in our interviews.

ADVANCES BOX

- Plant SynBio is a distinct, vibrant, diverse, and growing field with huge potential for real-world engineering impact that branched off mainline micro­bial SynBio but remains integrated with it.
- Plant SynBio was much influenced by the electronic engineering paradigm of the 2000s but has seen its shortcomings and is now hammering out plant-centric tools and ways to think and work.
- Plant SynBio is starting to internalize the essential differences between science (in which most plant SynBio practitioners were trained) and engineering (in which they were not trained).

### In the beginning

Although the first use of the term “synthetic biology” in its modern sense can be dated to 1980 (Hirschi et al. 2022), a landmark *Nature* paper in 2000 on construction of a genetic toggle switch in *Escherichia coli* (Gardner et al. 2000) is generally seen as the start of the SynBio field. It is worth noting that this seminal work came out of Professor Jim Collins’ group in the Biological Engineering Department at MIT, was done with a microbe, was based on an electronic engineering paradigm, and was funded by the Office of Naval Research. Associations with engineering departments, with electronic concepts instantiated in microbes, with elite universities, and with defense agency support were brand features in SynBio's early development, as discussed further below. The same associations remain strong to this day, although less in plant SynBio than in other areas. Also still strong is the early connection between SynBio and biomimetic chemistry, particularly in the branch of SynBio that seeks to redesign life from artificial building blocks (Benner and Sismour 2005). As artificial life-themed SynBio has little to do with plants we do not cover it in this review. The inception and progress of mainstream (i.e. microbe-centric and engineering-oriented) SynBio is well covered in many reviews, of which the following is an illustrative selection (de Lorenzo and Danchin 2008; de Lorenzo 2017; Erb 2019; Cameron et al. 2014; Clarke and Kitney 2020; Hanczyc 2020; Voigt 2020; Gallup et al. 2021; Volk et al. 2023).

### Hallmark principles and vocabulary of SynBio

SynBio has some distinguishing concepts and language. The first of these features—inherent in all forward engineering—is SynBio's prescriptive nature; this sets it apart from traditional descriptive biology (Calladine and ter Meulen 2012; Kurth et al. 2020; Hanson and de Lorenzo 2023). Other features have, over the years, almost become shibboleths (in-group catch phrases) used to set SynBio apart from all other biology; these include the principles of decoupling, abstraction, standardization, modularity, and orthogonality, and their fruits—automation and the indust­rialization of biology in biofoundry facilities (Endy 2005; Lucks et al. 2008; Chao et al. 2017). See the review by Liu and Stewart (2015) for definitions and explanations of these principles and the review by Holowko et al. (2021) for explanations of what biofoundries are and how they scale up throughput. Also diagnostic of SynBio are the words “platform” and “chassis,” which are metaphorical—and philosophically loaded—terms for the organism in which a SynBio project is executed (de Lorenzo 2011; Stewart et al. 2018; Voigt 2020).

**Figure 1.**
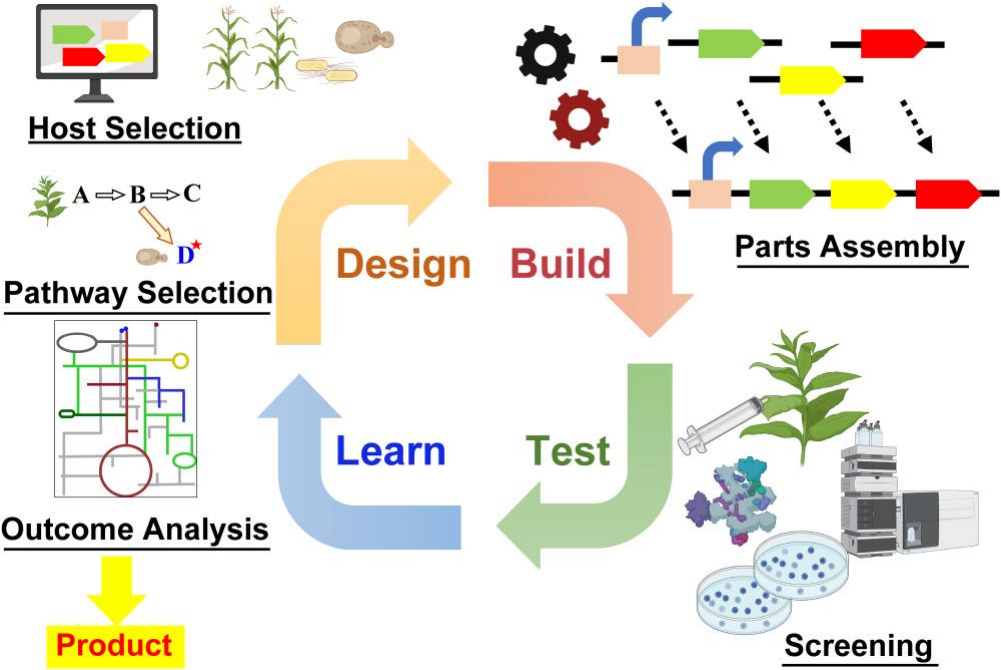
SynBio's iconic DBTL cycle. Note that the cycle outputs products, not just information and understanding.

Another SynBio hallmark is the design-build-test-learn (DBTL) cycle (Fig. 1). This iconic cycle conveys the idea that SynBio projects are iterative, i.e. that a design is first prototyped, then tested, and the test results inform further rounds of improvement until a final product exits the cycle (Poust et al. 2014). The idea of iterative cycles in design is not special to SynBio; it is in the ethos of all engineering (MacLeod 2012) and was explicitly adopted by metabolic engineering when it began (Bailey 1991).

What could also be considered a hallmark of SynBio is the porosity of its borders with other sectors of biology, most of all with metabolic engineering, but also with genetic engineering and, more recently, with genome editing. The continuum between metabolic engineering and metabolic SynBio projects is neatly captured in Fig. 1 of an excellent short review by Erb et al. (2017). This figure's message is that the more the enzyme reactions in a pathway and the pathway itself depart from nature, the more the project becomes SynBio, the culmination being new-to-nature pathways featuring novel reactions based on novel enzyme mechanisms. Plant-related examples of such enzymes and pathways are the late Arren Bar-Even's “daring metabolic designs” to replace Rubisco and the Calvin cycle with alternative reactions (Bar-Even 2018). Lastly, in practical terms, the continuum with metabolic engineering helped plant metabolic biochemists and engineers transition to SynBio (i.e. to “reinvent themselves”), as many of those working in mainline and specialized metabolism have done (Stewart et al. 2018).

## Plant SynBio's take-off and (future) flight path

### Oral history approach

As said above, SynBio's origin as a distinct field is usually dated to 2000 and began with gene circuits and then metabolic pathways in microbes, some of which involved parts—genes and enzymes—taken from plants (e.g. Chen and Weiss 2005; Ro et al. 2006). Plant SynBio sensu stricto (SynBio in which the target for improvement is a plant or photosynthetic bacterium, and what this review mainly covers) took off about a decade later, making it so young that it barely has any history under its belt. Moreover, plant SynBio's flight path to real-world engineering impact is still largely unclear. Unlike most areas of microbial SynBio, including engineering high-value plant products in yeast or bacteria (Schempp et al. 2017) and engineering plant-associated bacteria (Ke et al. 2021), plant SynBio does not really even have a flight *plan* yet, much less a flight *path*. The nearness to the present and the fluidity of everything about plant SynBio sensu stricto plus its lack of tangible impacts so far led us to decide not to survey the field and attempt an analytical history but instead to give readers a sense of what the early days of Plant SynBio were like through the eyes of representative founders of the field, i.e. to offer a pilot oral history. That we are not historians and did not ourselves witness plant SynBio taxiing along the runway before takeoff also played into this decision. For surveys of current activities and frontiers in plant SynBio, we refer readers to special journal issues (e.g. Benning and Sweetlove 2016; Stewart et al. 2018; Hanson et al. 2019; Kumar and Van de Peer 2020; Patron and Burgess 2023; Yang and Reyna-Llorens 2023), and to the reviews in Supplemental Table S1.

**Figure 2.**
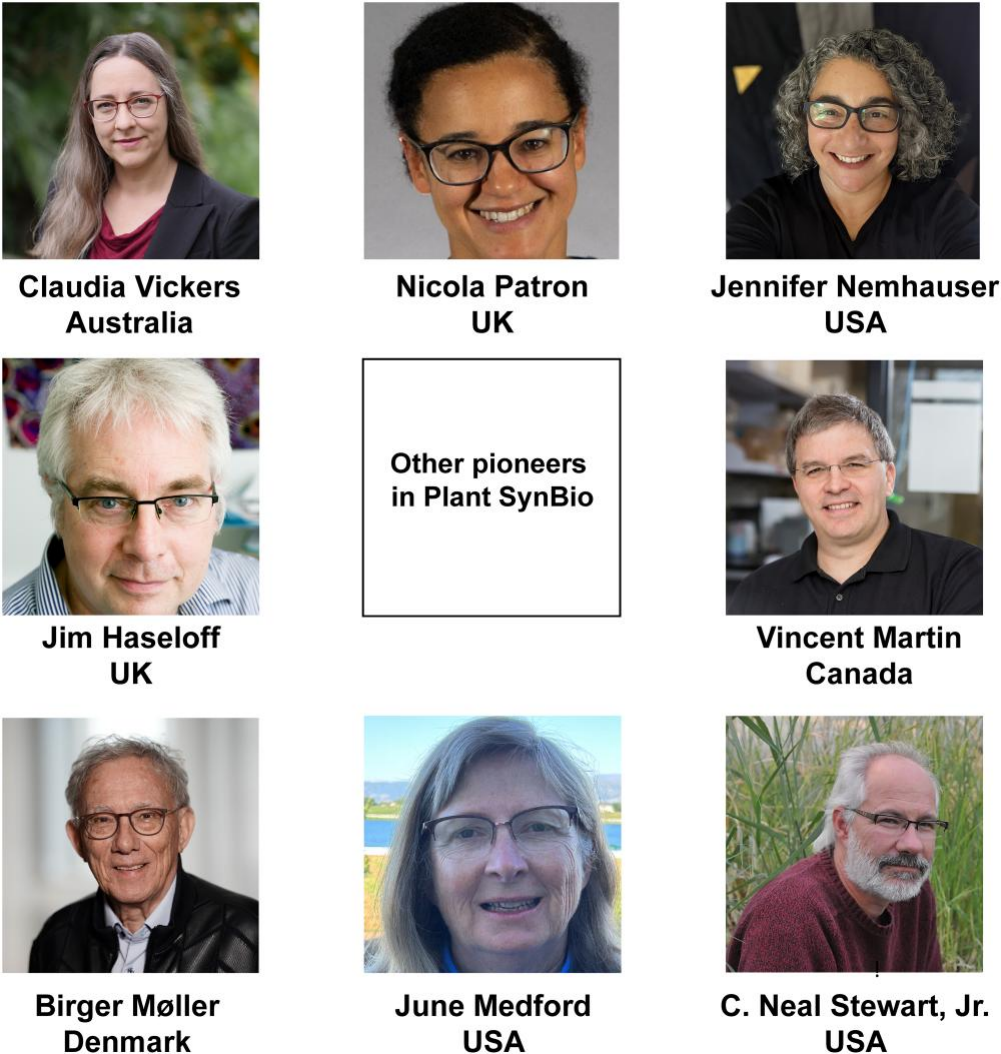
The 8 founding figures in plant SynBio who were interviewed. Box 1 gives microsketches of their careers and contributions to the field (arranged in clockwise order, starting with Claudia Vickers at the top left).

**Figure 3.**
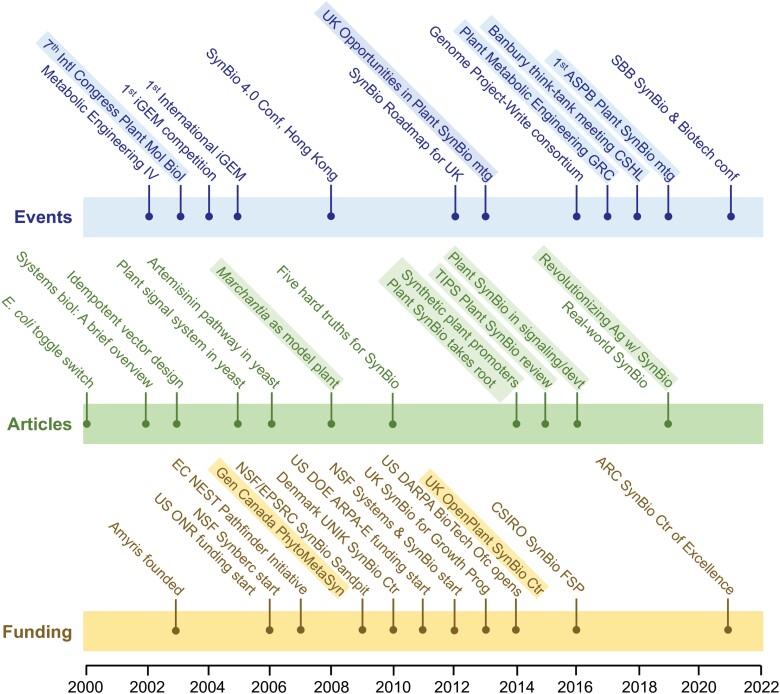
Timelines of selected meetings or other events, published articles, and funding programs that founders identified as pivotal in plant SynBio's evolution. Plant SynBio-specific entries are highlighted. That over half the entries are not from plant SynBio but from mainstream microbial SynBio reflects how much this mainstream inspired plant SynBio intellectually and nurtured it practically. Some dates are approximate. Timeline entries are documented in Supplemental Table S2.

We interviewed 8 founding figures who work in 5 of the countries that were among the first to see the potential of SynBio in general and plant SynBio in particular, and to put substantial funding programs in place. Figure 2 introduces these founders, and Box 1 sketches some of their key contributions to plant SynBio. The vacant central space in Fig. 2 symbolically honors the many other founders to whom the field owes much but whom we could not interview in the scope of this project. This applies particularly to plant researchers in Asia and other continents that were not among the very first to move into SynBio but that have contributed greatly to its subsequent flourishing. The interviews were one-on-one, via Zoom. We asked interviewees for their recollections and reflections on a standard set of topics, some of which were open-ended (Box 2 and Outstanding Questions Box). The interview recordings are available in Supplemental Appendix S1. We integrated the interview data into a set of timelines covering pivotal events, articles, and funding programs (Fig. 3 and Supplemental Table S2) and extracted key points from responses on the open-ended topics. Below, we discuss trends and highspots in the timelines and summarize key points from the responses. What else interviewees said on these and other points is in the recordings, which we encourage readers to visit; they are rich in experience, wisdom, and vision—and never boring! The timeline entries in Fig. 3 are partly based on consensus and are not attributed to particular founders; the text section on responses includes attributions. Lastly, a disclaimer: as an oral history based on the lived experiences and perspectives of a small number of people, this review could not be comprehensive or balanced in every aspect. We take full responsibility as authors for the review's shortcomings and ask readers to pardon these.

Box 1. **Brief biodata for the interviewees**

**Dr Claudia Vickers** has a background in plant molecular biology and engineering and has worked in research and development in universities, government, and industry. As Director of CSIRO's Synthetic Biology Future Science Platform from 2017 to 2022, she took a leading role in fostering development of SynBio in Australia and internationally, at scientific and strategic policy levels, serving as President of Synthetic Biology Australasia and as a founding member of the Global Biofoundries Alliance executive, and cochairing the WEF Synthetic Biology Global Future Council. Her recent research has been in developing biobased industrial products. She now leads her own venture, BioBuilt Solutions.

**Dr Nicola Patron** is a plant molecular biotechnologist and a national and international leader in plant SynBio. She directs the Earlham Biofoundry, one of the United Kingdom's first foundries and the only one specializing in plant SynBio workflows. She has taken leadership initiative to standardize DNA parts for the plant SynBio community. Her research applies SynBio approaches to understand how phenotypes emerge from network functions and to explore and utilize plant metabolic diversity. Through the creation of cutting-edge plant SynBio tools at the intersections of engineering, computing, biology, and design, she represents the future of SynBio and is an inspiration for diversity in STEM.

**Dr Jennifer Nemhauser** is a developmental biologist who builds SynBio tools to investigate and manipulate plant signaling dynamics, particularly those centered on auxin and other hormone signaling. Her ultimate goal is to uncover core principles that shape multicellular behaviors across eukaryotes. In addition to her scientific research and advocacy, her voice also reinforces the importance of inclusivity and diversity in the plant SynBio community and beyond. She has been a driving force in encouraging greater representation and equal opportunities for individuals from minoritized communities in STEM, as well as calling for major shifts in the culture of plant science toward equity-centered policies and actions.

**Dr Vincent Martin** has a microbiology background. He entered the nascent SynBio field as a postdoctoral at UC Berkeley in Jay Keasling's group, where he worked with a group of chemical engineers and cofounded Amyris Inc., which was established to commercialize production of the anti-malarial drug artemisinin in yeast and became a shining SynBio success. He went on to establish the first Synthetic Biology Center in Canada, equipped with a state-of-the-art Genome Foundry to accelerate the DBTL cycle. His research interest is in green biofactories for production of chemicals, pharmaceuticals, and next-generation therapeutics; he is currently working on natural products and organic acid synthesis in yeast.

**Dr C. Neal Stewart, Jr** is a plant biotechnologist (and songwriter! ) with many accomplishments and patents, particularly in crop genetic engineering. He has successfully developed transformation technologies for diverse plants. He became a trailblazer and early adopter of SynBio in plant biotech and agriculture and established University of Tennessee as one of the first US Land-Grant Institutions to take a lead in the field. He currently codirects the University of Tennessee's Center for Agricultural Synthetic Biology. He is an active proponent of plants as SynBio chassis. His current research ranges from agricultural sustainability to bioenergy crops and plants as environmental sensors.

**Dr June Medford** has a deep background in plant molecular biology and genetic engineering, having been in the Monsanto group that first commercialized transgenics. A very early entrant into plant SynBio, she rose rapidly to become an acclaimed world leader and advocate for SynBio in the plant sciences. She developed some of the first Plant Sentinels, featuring computationally designed sensors and control circuits to detect explosives, hazardous chemicals, and environmental pollutants. Recently, she has worked on a plant synthetic desalination genetic circuit. She has been instrumental in shaping plant SynBio's vision and advancing plant SynBio in government and industrial policies.

**Dr Birger Lindberg Møller** is a plant biochemist, particularly in the specialized metabolism area. He was among the first to recognize SynBio's potential in plant science and biotechnology, and quickly became a SynBio pioneer and visionary advocate for plant SynBio in Denmark, Europe, and worldwide. He leads the University of Copenhagen's Center for Synthetic Biology, whose mission is to develop knowledge-based solutions for grand challenges such as energy supply, transitioning to green growth, and advanced medical solutions. His research emphasizes harnessing chloroplasts as powerhouses for light-driven synthesis of natural products and bulk compounds, thus reducing reliance on fossil carbon.

**Dr Jim Haseloff** has a background in engineering and plant biology. He initially pioneered artificial RNA enzymes with targeted substrate specificity. An early SynBio adopter and leading SynBio expert, he quickly moved to the forefront of applying SynBio approaches to plants. His contributions include pioneering new quantitative imaging techniques, genetic circuits for cell-to-cell communication, and advancing the liverwort *Marchantia polymorpha* as a simple model system for bioengineering, for which he has developed DNA tools. He works through the OpenPlant initiative and Biomaker project to provide platforms for accessible technologies, fostering interdisciplinary activities and bringing together biologists, engineers, and social scientists.

Box 2. **Interview topics**

Interviewees were asked for their recollections and reflections on:

- Key meetings, workshops, or other events in plant SynBio development.
- Key funding agencies and programs and the dates they started.
- Two or three landmark papers or reviews that impacted the field overall, or them personally.
- Researchers whom they saw as founders.
- The suitability of the electronics engineering paradigm.
- Where plant SynBio *is* going—and where it *ought to be* going.
- Meetings, government initiatives, and industry collaborations that will be taking place soon to support the growth of plant SynBio.

### Timeline trends and highspots

Comparing the events and articles timelines with the funding timeline (Fig. 3) shows that the first 2 tend to have gaps in the middle and that much of the activity in the funding timeline comes in that middle region. Given that the funding timeline refers to when agencies *first started* funding SynBio (Box 2), a pattern emerges: influential meetings and papers in the early 2000s (not on plants) sparked interest inside funding agencies and among a few plant scientists. After lags of various lengths, the agencies started funding programs that were first microbe-centric and then extended to plants and mammals, and some plant scientists became early adopters. Plant-centric SynBio meetings and key papers then followed, about a decade behind the field as a whole. The lag between SynBio's inception and the start of major funding was notably short among the US funding agencies, particularly the Office for Naval Research and the Defense Advanced Research Projects Agency (DARPA), i.e. defense agencies, and the Advanced Research Projects Agency-Energy. These agencies remain major players, and all fund sensu stricto plant SynBio research and development. Although interviewees did not comment on this, defense agency support for plant SynBio has not been without controversy. One instance is DARPA's 2016 Insect Allies program to use insect dispersion of genetically modified viruses engineered to edit crop chromosomes in the field (Reeves et al. 2018).

A second trend evident in the timelines is that the earliest key SynBio papers and events, like the archetypal toggle switch paper (Gardner et al. 2000) and the first International Genetically Engineered Machine competition, came out of engineering departments in elite, globally networked institutions in the Northeastern United States and California; those identified in Fig. 3 are MIT, Princeton, and U.C. Berkeley; others include Harvard, Stanford, and Caltech. Although none of the earliest SynBio practitioners at these institutions were plant scientists, some became involved in plant-related work and helped inspire current and future plant scientists; MIT's Chris Voigt (e.g. Geddes et al. 2015) and Berkeley's Adam Arkin (Rubin et al. 2016) are examples. Much inspiration of this sort came out of the US National Science Foundation (NSF)-supported Synthetic Biology Engineering Research Center (SynBERC). This center, based at UC Berkeley and led by Jay Keasling (Synberc 2019), became a focal point for SynBio's spread into plant science.

A third trend concerns which countries outside the United States were nimblest in setting up government funding, research centers, and training programs for SynBio in general and plant SynBio in particular. In Europe, the U.K. and Denmark were particularly quick off the mark. Up to 2009, the United Kingdom was the only European country with an established SynBio funding scheme (Pei et al. 2012), and in 2009 the Danish Ministry of Research launched the UNIK SynBio initiative (Center for Synthetic Biology 2012). Further evidencing early leadership, in 2009 the U.K. Engineering and Physical Sciences Research Council (EPSRC) held a SynBio “Sandpit” jointly with NSF to address grand challenges (NSF 2008). Plant SynBio was represented at the Sandpit. Max Planck Institutes in Germany were strong centers. Beyond Europe, Australia was quite quick to systematically foster SynBio research and development (CSIRO 2022).

The publication and event highspots that interviewees identified necessarily had individual flavors; not everyone went to the same meetings or read the same things. There was nonetheless a fair amount of consensus on 2 points. First, early SynBio-related articles now held up as icons such as the first and fifth from the left in Fig. 3 truly did change mindsets in the way their citation statistics suggest they did. Second, the sense of a like-minded community born and nurtured in what were often quite small meetings was critical to get plant SynBio out of the construction hangar and onto the runway.

### Responses to open-ended questions

#### Researchers named by interviewees as founding figures

Supplemental Table S3 lists these researchers alphabetically. Europe, Australia, and Canada are strongly represented, particularly for plant SynBio as opposed to microbial SynBio, testifying to plant SynBio's international character. That founders of the whole SynBio field such as Frances Arnold, Chris Voigt, Jay Keasling, Adam Arkin, Tobi Erb, and Victor de Lorenzo appear in this list reflects not only their general influencer status via publications and talks, but also specific contacts and collaborations with several of the interviewees, in some cases via Synberc (see above).

#### Suitability of the electronics engineering paradigm

Like Victor de Lorenzo (de Lorenzo 2011), Antoine Danchin (Danchin 2012), and Jamie Davies (Davies 2019), all the founders interviewed saw the reigning electronic engineering paradigm—“cells as circuit boards”—as a double-edged sword: useful but of limited applicability, and even conceptually dangerous. The lively individual responses (edited from notes and transcripts) were as follows.

Nicola Patron: The analogy to electrical engineering is really helpful to think about things in a new way. But beware of the analogy because it will fall down really quickly. If you flog this analogy to everything in biology, it's definitely going to break. This is not the idea. Plants are not electronic circuits.

Vincent Martin: I think this analogy has a role to play but we have to be careful of the limitations, e.g. a genetic switch built with a transcription factor and a transcription factor-binding site. The premise would be that that the binding factor only binds to that sequence, and only binds in a very defined way. This is theoretically on/off, but there's a lot of data now coming out showing that transcription factors bind in a lot of different places and they bind to varying degrees to show varying effects. So the idea of being able to have orthologous or isolated circuits in the long run is going to be very, very difficult to do. But that being said, there are still wonderful examples of how these approaches can be used to control metabolism and cell development. So theoretically, it seems that it could be done. Practically, there are examples of successes. Whether or not those successes can scale to more complex systems or more advanced processes, I don’t know.

Claudia Vickers: It's a very useful framework as long as you recognize the limitations in the context of biological systems and the additional limitations in the context of plants. As long as you don’t stand up and say this is how it works, we have transistors and AND/OR/NOT gates that are always reproduced for X, it's fine. It's what we’re aiming for and is an objective to aim for as long as you recognize the challenges that are inherent in it and, in particular, as long as you understand the reasons behind why it's not that black-and-white, why it's not simple, then it's a very valuable paradigm. Because what you seek to achieve using those approaches is important, right? But if you pretend that it actually works that way and if you don’t recognize the limitations, then you will fail.

Jim Haseloff: I kind-of agree with the paradigm at one level. If you’re trying to put DNAs together, then having modular components that will be easy to string together, and where the relationships between gene, encoded protein, and target sequence are predictable, then there are a lot of useful principles that you can take from electrical engineering. But there are some people who take the electronics analogy to mean that you can deal with biological components as the equivalent of electronic components and draw circuits and make connections that way. You can probably do this at a local scale but, broadly speaking, I think the principles that build biological systems are very different to more-linear electronic systems. There are obviously examples of feedback and other network-type interactions in electronic systems. But *all* the interesting aspects of biological systems are based on networks and more social ways of interacting that couple into self-organization and self-repair. I think one has to recognize that the architecture of biological systems is fundamentally different to most human-engineered systems.

Jennifer Nemhauser: Metaphors are very powerful and it's also important to remember that they are metaphors. People who are trained as engineers really want biology to fit into that paradigm because it makes biology a lot less messy, which is definitely what engineers by training and by personality do. As biologists, our job is to hold complexity as a value to be comfortable with, to acknowledge when we’re reducing something to something simple, that we’re intentionally making assumptions. We can then remember those assumptions, and add back the complexity in a thoughtful way to challenge the conclusions we’ve come to. If someone working in plant SynBio finds a particular engineering paradigm useful for helping them think about how to approach their system—great. If not, they can try to find something else that feels more aligned.

Neal Stewart: I think it's a good example of a boilerplate to think about engineering plants, as circuit boards, as devices. But for real biological devices it kind-of breaks down, though. The idea here is that SynBio should be very useful for actually solving real-life problems, and the complexity of gene expression means that “cells as circuit boards” isn’t.

June Medford: As a paradigm, it helps to move us forward if we first start out and say, hey, can we make these simple gates? And yes, I would totally agree with a lot of folks who are coming from metabolic engineering and say it's much more complex. Absolutely. But if you don’t take it apart and put it in simple steps that your mind can understand, you’re never going to get to more complex issues. It is a thinking paradigm to move us forward; eventually we’re going to understand much more complex things like the influence of chromatin and epistasis that is so complex. We’re going to get there, but we need steps to get there. You don’t just go from “poof!” You have to take it in steps.

Birger Møller: This paradigm is helpful and unhelpful at the same time. It is a helpful conceptual frame—but biological units are not Lego bricks. Many pathways are organized in dynamic metabolons based on complex interactions between the modules. This serves to secure essential metabolic channeling. In this context, it is important to work with and learn from Nature. This is the way to build and scale up robust sustainable production systems for the future. Engineered regulatory circuits without context to Nature may work in the lab, but are not likely to be robust enough to work in field conditions.

#### Where plant SynBio is going—and where it ought to be going

Responses on these topics ranged from the grand strategic level through to operational and tactical levels (MacGregor 1992) and naturally tended to reflect each interviewee's domain expertise and interests. Where plant SynBio ought to be going (or the converse, where it is going but should not be) drew the most comments. The responses were pithy and thought-provoking, and converged on some common themes. Regarding where plant SynBio is currently going, there was a fair consensus that sustainability (particularly in agriculture) and climate change are major current directions. Regarding where it ought (or ought not) to be going, the idea that plant SynBio should “get real” or “come down to earth” came out in various ways, including statements that echo long-standing reservations (e.g. Good and Bell 1980) about the prospects for biofuels. A need for plant SynBio to break with entrenched, traditional ways of thinking and of doing things, and to look outward and engage with the world were other common threads. The responses below are preceded by “Is” or “Ought” in parentheses to make clear which category they refer to.

Nicola Patron: (Is) It is really exciting to see model-informed design of biological processes then getting them into the field to see how they work there happening, and to see trait engineering papers. (Ought) I did expect to see more fundamental biology taking in SynBio approaches.

Vincent Martin: (Is) Genomics is empowering enzyme discovery; the old model of “discover one enzyme → install in microbe → find product” is being overtaken, I don’t think this is where it's going. (Ought) Where I think it is going is our capacity to do 2 things: to bring these things to scale if they’re going to bring any value to anybody other than academic publications, but also our capacity to generate either natural or non-natural diversity at a much bigger scale than we can do now. We need to diversify the structures that we can produce. At present we are nowhere near matching nature's ability to do this. We need a combinatorial, high-throughput approach.

Claudia Vickers: (Is) Plant SynBio is going into climate change and sustainable agriculture. (Ought) We need to ask what impact SynBio can have that is *scalable* and will have impact at the planetary scale. Two areas that will be most impactful are in food and agriculture and there will be lots of plant SynBio applications that will be important for that. Examples are biobased construction materials and biobased binders. The holy grail of accelerating photosynthesis for carbon sequestration has proven to be very challenging. Plant SynBio should go where we can have planetary impact. There are many, many things, but the first thing you should do is OK, let's do an emissions analysis and a techno-eco­nomic analysis and determine is it scalable? Is it fast enough? And will it make a difference?

Jim Haseloff: (Is) “Plant blindness” is a problem. There is undue emphasis on microbial SynBio and new forms of bioproduction, and ignorance of the importance of plant systems and agriculture. I always find this quite curious because it's completely at odds with current practices in human culture, where agriculture is universal and cheap and has a huge capacity for production. Why is there so much emphasis on microbial-based systems, or in vitro-based systems, compared to the obvious importance, for the future, of plant-based SynBio and engineering biology? There is a bias in the plant world from the conservative (for good reason) agendas of large agribusiness companies that contrasts with startup culture for fermentations. Inari Agriculture and Afingen are exceptions. (Ought) For biofuels, even just simple life cycle analysis and economic analysis will tell you this is based on very doubtful premises. A lot of the early decision-making in this area was somewhat corrupted by the politics of bioethanol and energy security; there wasn’t really any solid scientific basis to it. With AI-protein folding we can now begin to predict consequences of residue changes in terms of interactions. Up to now, we have confused our (in)ability to understand these systems with the ability to begin engineering them. (See quotes on engineering in the Introduction.) Engineering development via gene regulatory circuits is hard because the circuit must be robust to environmental insults. This could be achieved by integrating environmental inputs to compensate for changes. Neo-organogenesis (building new plant organs) can be combined with metabolic engineering to locate pathway products in a new organ. This can also be the basis for neo-domestication (creating new crops).

Jennifer Nemhauser: (Ought) We need an ethos and genuine commitment to open-source, to DIY, to coproduction of knowledge, to novices and experts having high-quality conversations to find solutions for the food and climate crisis. A plant SynBio Reddit board where researchers can have conversations like that, to build a community up organically? We also need a more inclusive approach to build relationships with indigenous communities, and to train more international communities to find solutions for the food and climate crisis. There's a huge amount of biases left in the grant-reviewing process—there's still more work to do to be done around the people who are reviewing grants, maybe more than the people who are managing the funding. A more cross-disciplinary approach is needed.

Neal Stewart: (Ought) Plant SynBio should focus more on the chloroplast genome, which is more prokaryote-like, more amenable to manipulation. “Kill” (neutralization) switches are needed for containment. We should distinguish between SynBio toys and useful tools. Systems biology is delivering an endless parts list without understanding of how the parts fit together, still less how to manipulate them. We’re drowning in uncharacterized parts.

June Medford: (Is) Plant SynBio going towards sustainability of life on earth, we’re going to engineer a much more sustainable future! (Ought) ASPB should take more leadership. We should bring in big agribusiness players, e.g. Cargill, Bayer, and Syngenta. We need to help grant program managers to understand our plant SynBio needs and challenges, e.g. that plant research needs more time, so five-year rather than three-year funding.

Birger Møller: (Ought) Biofuels are “not an attractive climate measure” according to the Danish Council on Climate Change, which now “advises against investing in them as a means of achieving the climate goals” (Klima 2023). Two drivers for biofuels enthusiasm are that importing biomass is a way to offshore energy demands while still keeping a green façade and that enzyme companies have a vested interest in biomass valorization. It is important to build a startup culture. Promising research areas to pursue are (i) light-driven production in algae of high-value compounds with the pathways being expressed in the plant powerhouse, the chloroplast; (ii) windmill or solar cell electrically powered CO_2_ reduction to acetate and use of the acetate as a supplemental substrate for algal growth under light-limit­ed conditions in environmentally contained algae farms offering dual production of bulk and high-value products (Sørensen et al. 2022); and (iii) exploiting multifunctional P450s as new ways to make high-value molecules hard to obtain by classical chemical synthesis, e.g. triptonide (Hansen et al. 2022) and ginkgolides (Forman et al. 2022).

#### Meetings, government initiatives, industry collaborations that will support plant SynBio

Although this topic elicited fewer specific responses than the others, there was general satisfaction at how plant SynBio is gaining traction on the world stage (captured in the first quote below). Two interviewees cited regulatory policies as choke points.

Vincent Martin: Right now, there's so much happening. SynBio meetings of various types are now mushrooming all over the world, SynBio is spreading out fast from the Northern California and Boston areas where it used to be concentrated. I love the stuff that's coming out of DTU in Denmark. There's a lot of interesting things out of Australia. SynBio has really taken off in Asia. It's just wonderful, investigators are coming from all over the place as opposed to the field being dominated by a few groups.

Claudia Vickers: The Organization for Economic Co-operation and Development (OECD) Global Forum on Technology in Paris in June 2023 (“Shaping our future at the tech frontier”) featured SynBio; I contributed. We need to work on agile regulatory policy that can be updated as technology develops. We need to support startups, tech transfer, scaleup and to break through the issues of conflict-of-interest management, risk aversion, and intellectual property (IP) ownership that complicate getting technologies out of research institutions. We need dramatically more support for role models who demonstrate that such things are possible.

Neal Stewart: Government regulations are getting more precautionary, deeper—it's now a regulatory quagmire. Big Ag has been rather slow to embrace the power of SynBio, other than gene editing. Thus, industry is not in the lead (unlike it was for transgene technology), nor are government laboratories. It's therefore up to universities. More diversity in the funding pool is needed. DARPA still dominates. I wish we had a better name than “synthetic biology,” but I don’t know what to call it. I think there's a segment of the population who feel or have felt that biotechnology is maybe “playing God.” I’ve never really thought about it in those terms, because I feel that we are simply rearranging some of God's creation, in a very minor way, actually. I think we’re utilizing that and recombining it to a degree, to actually do something useful—which is what humans do. They innovate on top of innovation. Designing a “synthetic crop” of some sort might be what biologists in a hundred years end up doing. And here again, I don’t think it's “playing God.” I think it's just being creative as humans are creative.

Birger Møller: There's a lot of transfer of money to the plant field in Denmark and the Green Transition, whatever this means. Likewise, a few big players decide how the real moneys are spent. Now, SynBio is a buzzword, and everyone suddenly has been doing SynBio their entire life. We should really make sure that funds assigned to SynBio research are spent in the right way, so that we don’t have too many mishaps and the technology misses and people start not trusting what is promised.

## Take the SynBio tide at the flood

**Figure 4.**
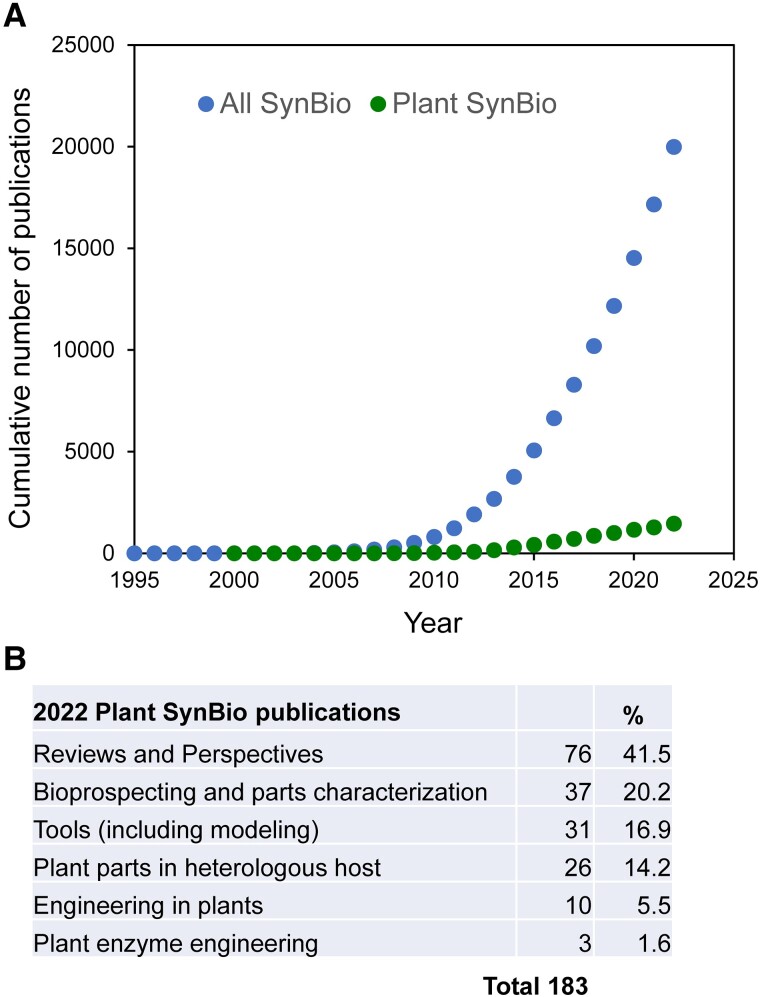
Trends in publications in SynBio as a whole and in plant SynBio **(A)** and a breakdown of 2022 plant SynBio publications by category **(B)**. Plant SynBio publications in 2022 were 7.3% of the total. Data were extracted from PubMed using the “All Fields” setting and the search terms “synthetic biology” or “synthetic biology” plus “plant.” The plant SynBio publications were curated manually.

A palpable feeling of being on the edge of a paradigm shift in Thomas Kuhn's deep original sense (Kuhn 1962), and even on the edge of history, pervaded our interviews. The feeling was more intense in some interviews than others, but always present. The Kuhnian paradigm shift is to a new, *actionable* way to think about and do bioscience. The historical aspect is that putting actionable SynBio ideas and information *to work* in the bioeconomy, agriculture, and the environment can help with many facets of the “global polycrisis” (Lawrence et al. 2022). The same awareness of being on the brink of something big comes through in the burgeoning plant SynBio literature, which in 2022 reached 7% of total SynBio articles (Fig. 4). Of the 2022 plant SynBio publications, >40% were forward-looking reviews or perspectives; ∼8% were original research articles on engineering plants or their enzymes. A 5-to-1 ratio of secondary to primary literature betokens a young field in the grip of a sense of promise.

We detected a second feeling, perhaps an anxious undercurrent, in the interviews. It was signaled by the length and strength of the responses about where plant SynBio *ought* and *ought not* to be going, and made manifest in Claudia Vickers’ insistence that “Plant SynBio should go where we can have planetary impact…the first thing you should do is…an emissions analysis and a techno-economic analysis and determine is it scalable? Is it fast enough? And will it make a difference?” The undercurrent of feeling was basically that time is running out if plant SynBio is to fully realize its potential to help fix the planetary problems that will change so much about life in the next 30 years.

OUTSTANDING QUESTIONS BOX

- How can the science-to-engineering culture shift from descriptive, largely qualitative, curiosity-driv­en biology to prescript­ive, quantitative, problem-solving SynBio be sped up and made easier?
- Which large-scale problems in agricultural, food, energy, and environmental syst­ems are key targets for SynBio, and how can SynBio technology be deployed to deliver workable solutions?
- How do we open SynBio researchers’ minds to the urgency of engaging with real-world problems while the chances of finding and being able to implement solutions are still optimal?

There was a somewhat similar sense in microbial SynBio a decade ago, expressed in a hard-hitting *Nature* article (“Five hard truths for synthetic biology”; Kwok 2010). This article quoted SynBio guru George Church as saying: “The field has had its hype phase. Now it needs to deliver.” A dozen years on, microbial SynBio still has plenty of hype (Hanson and de Lorenzo 2023), but it has real products too (Voigt 2020). “Hype” is too harsh a term for most current plant SynBio ideation. Maybe “distraction by misinformed popular narratives,” or “blue skies thinking unconnected to practical needs,” or “visions with a thousand-year timeline when we only have decades,” or even “aspiring engineers learning numeracy on the job” would be nearer the mark. In any case, these lines from Shakespeare's play *Julius Caesar* express the tension in our interviews between scientific excitement on one hand and the great but time-limited opportunity to take up real-world engineering challenges on the other.

*There is a tide in the affairs of [humans] which, taken at the flood, leads on to fortune;*

*Omitted, all the voyage of their life is bound in shallows and in miseries.*

*On such a full sea are we now afloat; And we must take the current when it serves,*

*Or lose our ventures.*

## Supplementary Material

kiad585_Supplementary_Data

## Data Availability

The data underlying this article are available in the article and in its online supplementary material.

## Supplemental data

The following materials are available in the online version of this article.

**Supplemental Table S1.** Recent reviews of plant SynBio.

**Supplemental Table S2.** Documentation of the entries in Fig. 3 plus entries that were not included.

**Supplemental Table S3.** Researchers named by interviewees as founding figures.

**Supplemental Appendix S1.** Recordings of the 8 interviews.

## Dive Curated Terms

The following phenotypic, genotypic, and functional terms are of significance to the work described in this paper:

NSF Gramene: AT4G04910
NSF Araport: AT4G04910
