# Supplementary material for: A pilot oral history of plant synthetic biology
Source: Plant Physiol. 2024 Jan 2;195(1):36–47. doi: 10.1093/plphys/kiad585 (PMC11060686; doi:10.1093/plphys/kiad585)
Supplement: kiad585_Supplementary_Data [file kiad585_supplementary_data.zip › PP2023TR01165R1_Supplemental_Data.pdf]

Supplemental Table S1. Recent reviews of plant SynBio.

Alamos S, Shih PM (2023) How to engineer the unknown: Advancing a quantitative and predictive understanding of plant and soil biology to address climate change. PLoS Biol 21: e3002190	https://www.ncbi.nlm.nih.gov/pmc/articles/PMC10351729/
Boehm CR, Pollak B, Purswani N, Patron N, Haseloff J (2017) Synthetic botany. Cold Spring Harb Perspect Biol 9: p.a023887	https://www.ncbi.nlm.nih.gov/pmc/articles/PMC5495061/
Brophy JA (2022) Toward synthetic plant development. Plant Physiol 188: 738–748	https://www.ncbi.nlm.nih.gov/pmc/articles/PMC8825267/
Goold HD, Wright P, Hailstones D (2016) Emerging opportunities for synthetic biology in agriculture. Genes 9: 341	https://www.ncbi.nlm.nih.gov/pmc/articles/PMC6071285/
Patron NJ (2020) Beyond natural: synthetic expansions of botanical form and function. New Phytol 227: 295–310	https://www.ncbi.nlm.nih.gov/pmc/articles/PMC7383487/
Rizzo P, Chavez BG, Leite Dias S, D'Auria JC (2023) Plant synthetic biology: from inspiration to augmentation. Curr Opin Biotechnol 79: 102857	https://doi.org/10.1016/j.copbio.2022.102857
Roell MS, Zurbriggen MD (2020) The impact of synthetic biology for future agriculture and nutrition. Curr Opin Biotechnol 61: 102–109	https://doi.org/10.1016/j.copbio.2019.10.004
Sargent D, Conaty WC, Tissue DT, Sharwood RE (2022) Synthetic biology and opportunities within agricultural crops. J Sustain Agric Environ 1: 89–107	https://doi.org/10.1002/sae2.12014
Wang L, Zang X, Zhou J (2022) Synthetic biology: A powerful booster for future agriculture. Advanced Agrochem 1: 7-11	https://doi.org/10.1016/j.aac.2022.08.005
Wang Y, Demirer GS (2023) Synthetic biology for plant genetic engineering and molecular farming. Trends Biotechnol 2023 Apr 1:S0167-7799(23)00088-4	https://doi.org/10.1016/j.tibtech.2023.03.007
Wright RC, Nemhauser J (2019) Plant synthetic biology: quantifying the “known unknowns” and discovering the “unknown unknowns”. Plant Physiol 179: 885–893	https://www.ncbi.nlm.nih.gov/pmc/articles/PMC6393784/
Wright RC, Neres DF, Chaisupa P, Bryant JA Jr (2023) Protein engineering and plants: the evolution of sustainable agriculture. The Biochemist 45: 12–17	https://doi.org/10.1042/bio_2023_101
Wurtzel ET, Vickers CE, Hanson AD, Millar AH, Cooper M, Voss-Fels KP, Nikel PI, Erb TJ (2019) Revolutionizing agriculture with synthetic biology. Nat Plants 5: 1207–1210	https://doi.org/10.1038/s41477-019-0539-0

Supplemental Table S2. Documentation of the entries in Figure 3 plus entries that were not included.

Abbreviated entry name from Figure 3	Documentation
Events	
Metabolic Engineering IV	AIChE Metabolic Engineering IV: Applied Systems Biology - Il Ciocco, Italy, 2002
7th Intl Congress Plant Mol Biol	https://www.cbd.int/kb/record/meeting/1248?Country=es
1st iGEM competition	https://competition.igem.org/previous-years
1st International iGEM	https://competition.igem.org/previous-years
SynBio 4.0 Conf, Hong Kong	http://bmi.ym.edu.tw/wp/wp-content/uploads/2008/06/synthetic-biology_40_flyer.pdf
SynBio Roadmap for UK	https://openaccess.city.ac.uk/id/eprint/16096/
UK Opportunities in Plant SynBio mtg	https://doi.org/10.1093/jxb/eru013
Genome Project-Write consortium	https://www.science.org/doi/10.1126/science.aaf6850
Plant Metabolic Engineering GRC	https://www.grc.org/plant-metabolic-engineering-conference/2017/
Banbury think-tank meeting CSHL	https://www.cshl.edu/wp-content/uploads/2018/12/PLANT18_Program.pdf
1st ASPB Plant SynBio mtg	https://blog.aspb.org/synthetic-biology-at-plant-biology-2019/
SBB SynBio & Biotech conf	https://easychair.org/cfp/sbb2021
Articles	
E. coli toggle switch	https://doi.org/10.1038/35002131
Systems biol: A brief overview	https://www.science.org/doi/full/10.1126/science.1069492
Idempotent vector design	http://hdl.handle.net/1721.1/21168
Plant signal system in yeast	https://doi.org/10.1038/nbt1162
Artemisinin pathway in yeast	https://doi.org/10.1038/nature04640
Marchantia as model plant	https://doi.org/10.1093/pcp/pcn085
Five hard truths for SynBio	https://doi.org/10.1038/463288a
Plant SynBio takes root	https://www.science.org/doi/full/10.1126/science.1261140
Synthetic plant promoters	https://doi.org/10.1111/pbi.12206
TIPS Plant SynBio review	https://doi.org/10.1016/j.tplants.2015.02.004
Plant SynBio in signaling/devt	https://doi.org/10.1038/nplants.2016.10
Real-world SynBio	https://doi.org/10.3390/life9010006
Revolutionizing Ag w/ SynBio	https://doi.org/10.1038/s41477-019-0539-0
Funding	
Amyris founded	https://www.fastcompany.com/3000040/rise-and-fall-company-was-going-have-us-all-using-biofuels
US ONR funding start	https://www.nre.navy.mil/media/document/70th-lecture-series-flyer-voigt-011515pdf
NSF Synberc start	https://ebrc.org/synberc/
EC NEST Pathfinder Initiative	https://journals.sagepub.com/doi/10.1177/0963662510393624
Gen Canada PhytoMetaSyn	https://doi.org/10.1016/j.tibtech.2011.10.001
NSF/EPSRC SynBio Sandpit	https://www.nsf.gov/pubs/2009/nsf09012/nsf09012.jsp?org=NSF
Denmark UNIK SynBio Ctr	https://synbio.ku.dk/research-presentation/unik-synthetic-biology/
US DOE ARPA-E funding start	https://arpa-e.energy.gov/events/tool-development-transformational-biotechnology-advances-workshop
NSF Systems & SynBio start	https://new.nsf.gov/funding/opportunities/systems-synthetic-biology
UK SynBio for Growth Prog	https://www.ukri.org/what-we-do/our-main-funds-and-areas-of-support/browse-our-areas-of-investment-and-support/synthetic-biology-for-growth/
US DARPA BioTech Ofc opens	https://www.darpa.mil/about-us/timeline/bto-opens
UK OpenPlant SynBio Ctr	https://www.cam.ac.uk/research/news/openplant-major-boost-for-synthetic-biology
CSIRO SynBio FSP	https://research.csiro.au/synthetic-biology-fsp/
ARC SynBio Ctr of Excellence	https://www.coesb.com.au/

Entries not included in Figure 3	Documentation
Events	
2003 TERPNET Meeting, Lexington KY	
2009 SynBio Meeting: Mathematics & Biology	2009 Synthetic Biology Meeting: Mathematics and Biology, Ohio State University
2018 "Green" Exhibition at the SMK, Denmark	https://green-slsa2018.ku.dk/images/Green_Final.pdf
Articles	
Tunability and noise dependence	https://www.science.org/doi/full/10.1126/science.1137455
Encoding and decoding cellular information	https://doi.org/10.1016/j.cell.2013.02.005
Dual function of cytochrome P450 CYP76 family	https://doi.org/10.1104/pp.114.244814
Cultivating plant SynBio from systems biology	https://doi.org/10.1111/j.1469-8137.2008.02433.x
Synthetic eukaryotic signal transduction pathway	https://doi.org/10.1038/msb.2009.28
Programmable ligand detection in plants	https://doi.org/10.1371/journal.pone.0016292
Small molecule biosensors in eukaryotes	https://doi.org/10.7554/eLife.10606
Direct light-driven biosynthesis	https://doi.org/10.1021/acschembio.6b00190
Funding	
Denmark bioSYNergy SynBio Project	https://synbio.ku.dk/news/synbio_2016/
Max Planck Research Network for SynBio	https://www.maxsynbio.mpg.de/home
UQ Dow Centre for Sustainable Engineering	https://chemeng.uq.edu.au/dow-centre-sustainable-engineering-innovation

Supplemental Table S3. Researchers named by interviewees as founding figures.
Researchers named by multiple interviewees are bolded; those whose work is plant-centric are highlighted in green.

**Name**	**Discipline**	**Institution**	**Country**
Frances Arnold	Enzyme engineering	Caltech	USA
Adam Arkin	Bioengineering	UC Berkeley	USA
Ian Baldwin	Chemical ecology	Max Planck Institute for Chemical Ecology	Germany
Joerg Bohlmann	Plant specialized metabolism	University of British Columbia	Canada
Michael Elowitz	Bioengineering	Caltech	USA
**Drew Endy**	Bioengineering	Stanford	USA
Victor de Lorenzo	Bacterial engineering	CNB-CSIC	Spain
Tobias Erb	Biochemistry and synthetic metabolism	Max Planck Institute for Terrestrial Microbiology	Germany
**Andrew Hanson**	Metabolic biochemistry & engineering	University of Florida	USA
**Jim Haseloff**	Plant growth engineering	University of Cambridge	UK
**Jay Keasling**	Metabolic engineering	UC Berkeley / JBEI	USA
Tom Knight	Computer engineering	MIT	USA
Toni Kutchan	Plant specialized metabolism	Donald Danforth Plant Science Center	USA
Radhakrishnan Mahadevan	Metabolic engineering	University of Toronto	Canada
Cathie Martin	Metabolic biochemistry & engineering	John Innes Centre	UK
**June Medford**	Genetic engineering	Colorado State University	USA
A Harvey Millar	Biochemistry	University of Western Australia	Australia
Diego Orzaez	Plant biotechnology	IBMCP, CSIC	Spain
**Anne Osbourn**	Plant specialized metabolism	John Innes Centre	UK
Ingo Potrykus	Genetic engineering	Swiss Federal Institute of Technology	Switzerland
Patrick Shih	Synthetic biology	UC Berkeley	USA
Surinder Singh	Metabolic engineering	CSIRO	Australia
Ian Small	Molecular genetics	University of Western Australia	Australia
Danièle Werck-Reichardt	P450 biochemistry	CNRS Strasbourg	France
**Chris Voigt**	Bioengineering	MIT	USA
Peter Waterhouse	Genetic engineering	University of Sydney / CSIRO	Australia
Ron Weiss	Bioengineering	MIT	USA
Eleanore Wurtzel	Metabolic biochemistry and engineering	Lehman College, CUNY	USA
Matias Zurbriggen	Optogenetics	Universität Düsseldorf	Germany
