# Supplementary material for: A pilot oral history of plant synthetic biology
Source: Plant Physiol. 2024 Jan 2;195(1):36–47. doi: 10.1093/plphys/kiad585 (PMC11060686; doi:10.1093/plphys/kiad585)
Supplement: kiad585_Supplementary_Data [file kiad585_supplementary_data.zip › KIAD585_Supplemental Appendix S1.pdf]

Supplemental Appendix S1. Recordings of the eight interviews.

Recordings are available at: http://academic.oup.com/plphys/article-lookup/doi/10.1093/plphys/kiad585#supplementary-data

Jennifer Nemhauser

June Medford

Nicola Patron

Claudia Vickers

Neal Stewart

Vincent Martin

Jim Haseloff

Birger Moller
